# Supplementary material for: A Universal Multi-Epitope Vaccine Design Against Porcine Reproductive and Respiratory Syndrome Virus via Bioinformatics and Immunoinformatics Approaches
Source: Vet Sci. 2024 Dec 16;11(12):659. doi: 10.3390/vetsci11120659 (PMC11680090; doi:10.3390/vetsci11120659)
Supplement: Supplementary file 1 [file vetsci-11-00659-s001.zip › vetsci-3319851-supplementary.pdf]

veterinary sciences

MDPI

Supplementary Materials

Table S1 Comparison of genomic and encoded protein identity among seven representative strains.

Item	VR-2332	LV	CH1a	JXA1	NADC30	NADC34	RFLP_1-4-4
Genome	100%	60.4%	91.5%	89.6%	85.2%	84.9%	85.0%
Nsp1α	100%	65.6%	98.9	96.1%	96.7%	95.6%	94.4%
Nsp1β	100%	43.2%	85.8%	84.3%	76.5%	77.9%	78.4%
Nsp2	100%	41.1%	84.3%	80.3%	75.8%	73.1%	71.9%
Nsp3	100%	51.9%	96.1%	95.7%	92.6%	92.6%	94.8%
Nsp4	100%	61.3%	96.6%	94.6%	92.7%	93.2%	94.6%
Nsp5	100%	96.0%	93.0%	92.4%	89.5%	88.3%	95.3%
Nsp6	100%	82.4%	94.1%	94.1%	94.1%	94.1%	100.0%
Nsp7α	100%	57.3%	97.3%	96.0%	93.3%	94.7%	92.0%
Nsp7β	100%	35.9%	84.7%	82.0%	79.3%	82.0%	83.8%
Nsp8	100%	69.6%	100.0%	100.0%	93.5%	93.5%	91.3%
Nsp9	100%	74.4%	97.8%	97.8%	95.9%	96.4%	96.4%
Nsp10	100%	65.2%	95.7%	95.7%	94.6%	95.7%	95.2%
Nsp11	100%	75.0%	94.6%	94.2%	95.1%	95.1%	94.2%
Nsp12	100%	44.4%	96.8%	95.5%	93.5%	90.3%	91.6%
GP2α	100%	63.7%	95.3%	93.0%	88.7%	88.2%	86.7%
GP2b (E)	100%	76.1%	90.5%	90.5%	87.8%	90.5%	87.8%
GP3	100%	59.6%	88.6%	85.5%	80.8%	82.7%	81.2%
GP4	100%	67.0%	90.5%	89.4%	86.0%	88.8%	85.5%
GP5α	100%	50.0%	87.2%	83.0%	93.5%	91.5%	89.4%
GP5	100%	59.3%	91.5%	88.1%	82.5%	86.1%	85.1%
M	100%	78.7%	97.1%	97.1%	93.1%	93.1%	93.1%
N	100%	63.6%	96.8%	95.2%	92.7%	91.9%	91.9%

Note: Sequence identity were analyzed after alignment by Clustal W method in MegAlign software.

Table S2 Predicted binding affinities of seven candidate Th epitopes with seven HLA molecules.

Protein	Epitope sequence	High binders	HLA-DRB1 03:01		HLA-DRB1 07:01		HLA-DRB1 15:01		HLA-DRB3 01:01		HLA-DRB3 02:02		HLA-DRB4 01:01		HLA-DRB5 01:01	
			**IC50**	**Rank**	**IC50**	**Rank**	**IC50**	**Rank**	**IC50**	**Rank**	**IC50**	**Rank**	**IC50**	**Rank**	**IC50**	**Rank**
Nsp9	RSTPAIVRWFAAHLLYELAC (H/N)	12	—	—	7.8	0.6	3.4	0.03	—	—	—	—	—	—	—	—
	PNRDRILAALAYHMKANNVSEYYASAAAILMDSCA (N/S)	28	—	—	5.7	0.3	6.1	0.17	11.9	0.6	10.5	0.8	—	—	3.2	0.12
	VQPLIVYSDDLVLYAES	9	17.8	0.6	—	—	17.1	1.2	19.7	1.1	—	—	—	—	—	—
Nsp11	WPDRLVASLRPIHKYSRA (A/T)	12	24.3	0.9	—	—	—	—	—	—	—	—	24.8	0.9	3.3	0.12
Nsp12	LACAEFSLDDPVRYKHTWGFESDTAYLYEFTG (R/K)	22	5.6	0.04	—	—	6.4	0.17	5.1	0.12	—	—	—	—	—	—
GP4	EFTQRSLVVDHVRLLHFMTPETMR	9	35.6	1.5	—	—	—	—	14.4	0.8	—	—	29	1.1	—	—
GP5	RYTNFLLDTKGRLYRWRSPVI (R/K)	8	6.1	0.05	—	—	—	—	5.1	0.12	—	—	—	—	—	—

veterinary sciences

Table S3 Predicted binding affinities of 65 initially screened CTL epitopes with seven SLA molecules.

Protein	Epitope sequence	Epitope Conservation	SLA-1:0401		SLA-1:0801		SLA-2:0101		SLA-2:0401		SLA-2:0502		SLA-2:1001		SLA-3:0401	
			Affinity(nM)	%Rank	Affinity(nM)	%Rank	Affinity(nM)	%Rank	Affinity(nM)	%Rank	Affinity(nM)	%Rank	Affinity(nM)	%Rank	Affinity(nM)	%Rank
nsp1a	RMTSGNLNF	6/7	394.62	1	369.82	0.1	1915.27	1	—	—	—	—	495.29	0.2	2904.96	0.8
	TVLKNLQVY	3/7	449.33	1	1542.59	0.8	1834.15	2	4084.68	—	—	—	—	—	—	—
nsp1β	RVEPNTSPL	5/7	164.27	0.5	—	—	—	—	—	—	—	—	—	—	—	—
nsp2	RTAPSEVAF	3/7	170.61	0.5	864.68	0.8	2122.61	1.5	—	—	—	—	—	—	—	—
	FTAALRSGY	2/7	88.66	0.25	635.24	0.4	947.98	0.17	1067.79	0.25	—	—	1297.38	1.5	—	—
	SSAAAIPPY	1/7	99.86	0.3	250.51	0.05	727.23	0.1	306.02	0.03	—	—	432.64	0.15	—	—
	MILETPPPY	3/7	274.64	0.8	346.57	0.07	—	—	1525.99	0.8	—	—	963.49	0.8	—	—
	FVAAVRCGY	1/7	135.94	0.4	681.52	0.5	1414.68	0.5	3911.67	1.5	—	—	—	—	—	—
	LTWRNTSAY	1/7	138.16	0.4	497.98	0.2	832.55	0.12	869.37	0.15	—	—	1262.76	1.5	—	—
	SVIPFRAPF	2/7	153.12	0.4	506.12	0.25	1593.49	0.8	—	—	—	—	788.71	0.8	—	—
	FTTALRSGY	4/7	164.27	0.5	1430.07	1.5	1304.42	0	2088.44	0.8	—	—	—	—	—	—
	ATVWTTSQF	2/7	168.78	0.5	557.89	0.3	—	1.5	—	0.8	—	—	—	—	—	—
	TTSQFYAHY	2/7	178.16	0.5	—	1	—	0.2	279.13	0.03	—	—	—	1.5	—	—
	TVWTTSQFY	2/7	181.07	0.5	—	0.3	—	0.3	—	—	—	—	—	—	—	—
	RMWDRVDML	6/7	—	—	—	—	—	—	—	—	—	—	333.69	0.07	—	—
nsp3	NVAGLVTPY	4/7	238.6	0.8	—	0.4	—	—	—	0.12	—	—	—	1.5	—	—
	VSLWLLGRY	5/7	—	—	—	—	—	—	611.63	0.07	—	—	—	—	—	—
nsp4	KLSELSEFF	6/7	1097.07	2	—	1.5	—	—	—	—	—	—	—	1.5	—	—
	SSMGSGGVF	6/7	—	1.5	323.04	0.07	—	—	—	0.5	—	—	—	0.3	—	—
nsp8	SVEQALGMM	3/7	375.87	1	—	—	—	—	—	—	—	—	—	—	—	—
	RIIDKLQGL	6/7	—	—	—	—	—	—	—	—	—	—	434.98	0.15	—	—
nsp9	GIDGTLWDF	5/7	27.26	0.07	—	2	—	—	—	—	—	—	—	—	—	—
	CAEEHLPSY	2/7	273.16	0.8	—	—	—	—	—	2	—	—	—	—	—	—
	YAQHMVLSY	5/7	464.16	1	461.65	0.17	—	0.25	—	0.8	—	—	—	0.8	—	—

	YSFPGPPFF	2/7	551.89	1.5	461.65	0.17	—	0.15	—	1	—	0.8	—	0.25	—	—
	KYDLSTQGF	7/7	123.32	0.4	—	—	—	—	—	—	—	—	—	—	—	—
	TIPASVLDY	3/7	144.27	0.4	—	—	—	—	—	—	—	—	—	—	—	—
	ALSGVTQGF	5/7	215.3	0.8	412.08	0.12	—	—	—	0.5	—	—	—	0.5	—	—
	RWFAANLLY	3/7	—	—	—	2	503.39	0.03	—	1.5	—	—	—	—	—	0.8
nsp10	ICDAIQPDY	6/7	51.06	0.15	—	—	—	—	—	—	—	—	—	—	—	—
	RARHAIFVY	6/7	—	—	—	—	—	0.17	—	2	—	—	—	2	277.63	0.05
	EVDLPDGDY	5/7	75.79	0.25	—	—	—		—	—	—	—	—	—	—	—
	MVNTTRVTY	5/7	113.1	0.3	—	0.8	—	0.5	—	—	—	—	—	2	—	—
nsp11	KVAHNLGFY	6/7	64.43	0.17	—	1	—	0.17	—	1	—	—	—	—	—	—
	VTSKYLPRF	5/7	236.04	0.8	—	—	—	—	—	1	—	—	—	—	—	—
	MVWKDKTAY	5/7	250.51	0.8	—	0.5	—	1	—	—	—	—	—	—	—	—
	LTDVYLPDL	4/7	454.22	1	—	—	—	—	—	—	—	—	—	—	—	—
nsp12	GADLAVTPY	6/7	47.08	0.15	—	1.5	—	—	—	—	—	—	—	—	—	—
	YQLASYASY	4/7	—	—	211.83	0.03	—	1.5	—	0.8	—	—	229.74	0.03	—	—
	FTWYQLASY	4/7	545.95	1.5	—	0.25	—	0.4	—	0.2	—	—	—	0.8	—	—
	RVPVNSTVY	3/7	—	1.5	—	—	—	2	—	—	—	—	—	—	—	2
GP2a	ASDWFAPRY	6/7	12.64	0.03	—	0.8	—	1.5	—	0.2	—	—	—	—	—	—
	AIEAETCKY	6/7	49.43	0.15	—	2	—	—	—	—	—	—	—	—	—	—
	SVAASCTLF	6/7	174.34	0.5	—	0.4	—	1	—	1	—	—	—	—	—	—
	ALPFTLSSY	2/7	260.18	0.8	—	0.8	—		—	—	—	—	—	—	—	—
	KYLASRLPM	6/7	—	—	—	—	—	1	—	—	—	—	—	—	403.25	0.05
E	FVDAFTEF	7/7	247.81	0.8	—	1.5	—	—	—	—	—	—	—	—	—	—
GP3	MANSCTLLY	1/7	135.94	0.4	365.84	0.1	359.95	0.01	—	0.3	—	1.5	—	2	—	—
	VVANSNATF	3/7	195.32	0.5	—	0.5	—	—	—	—	—	—	—	2	—	—
	LSFSYTAQF	6/7	—	—	—	0.8	—	—	570.09	0.07	—	—	—	1	—	—
	QVDGGNWFH	6/7	233.5	0.8	—	—	—	—	—	—	—	—	—	—	—	—
	YTAQFHPEI	6/7	—	—	—	—	—	1.5	—	—	216.47	0.03	—	—	—	—
	WLAFLSFSY	7/7	—	—	451.77	0.17	—	1.5	—	—	—	—	—	1.5	—	—

	FSFELTVNY	2/7	—	—	—	0.8	—	0.8	409.85	0.05	—	—	466.68	0.17	—	—
GP4	RTAIGTPVY	6/7	133.03	0.4	412.08	0.12	—	0.05	—	0.8	—	—	—	1	—	—
	LMLSSCLFY	6/7	—	—	466.68	0.17	—	0.17	—	—	—	—	—	2	—	—
	MLSSCLFY	6/7	296.25	0.8	576.3	0.3	—	0.5	—	—	—	—	—	2	—	—
	VVDHVRLL	5/7	339.15	0.8	—	—	—	—	—	—	—	—	—	—	—	—
GP5a	RQQQQQLPF	2/7	—	2	—	0.4	—	—	—	—	—	—	418.82	0.12	—	0.12
GP5	LLDTKGRLY	4/7	105.42	0.3	—	1.5	—	—	—	—	—	—	—	—	—	—
	ATVSTAGY	2/7	63.05	0.17	484.69	0.2	—	0.5	274.64	0.03	—	—	—	2	—	—
	TVSTAGFY	1/7	163.39	0.5	—	—	—	2	—	—	—	—	—	—	—	—
	AVETFVIF	5/7	264.44	0.8	—	—	—	—	—	—	—	—	—	—	—	—
N	LSDSGRISY	6/7	32.24	0.1	—	1	—	1	—	1.5	—	—	—	—	—	—
	RISYTVEF	6/7	247.81	0.8	—	1.5	—	—	—	—	—	—	—	—	—	—

veterinary sciences

MDPI

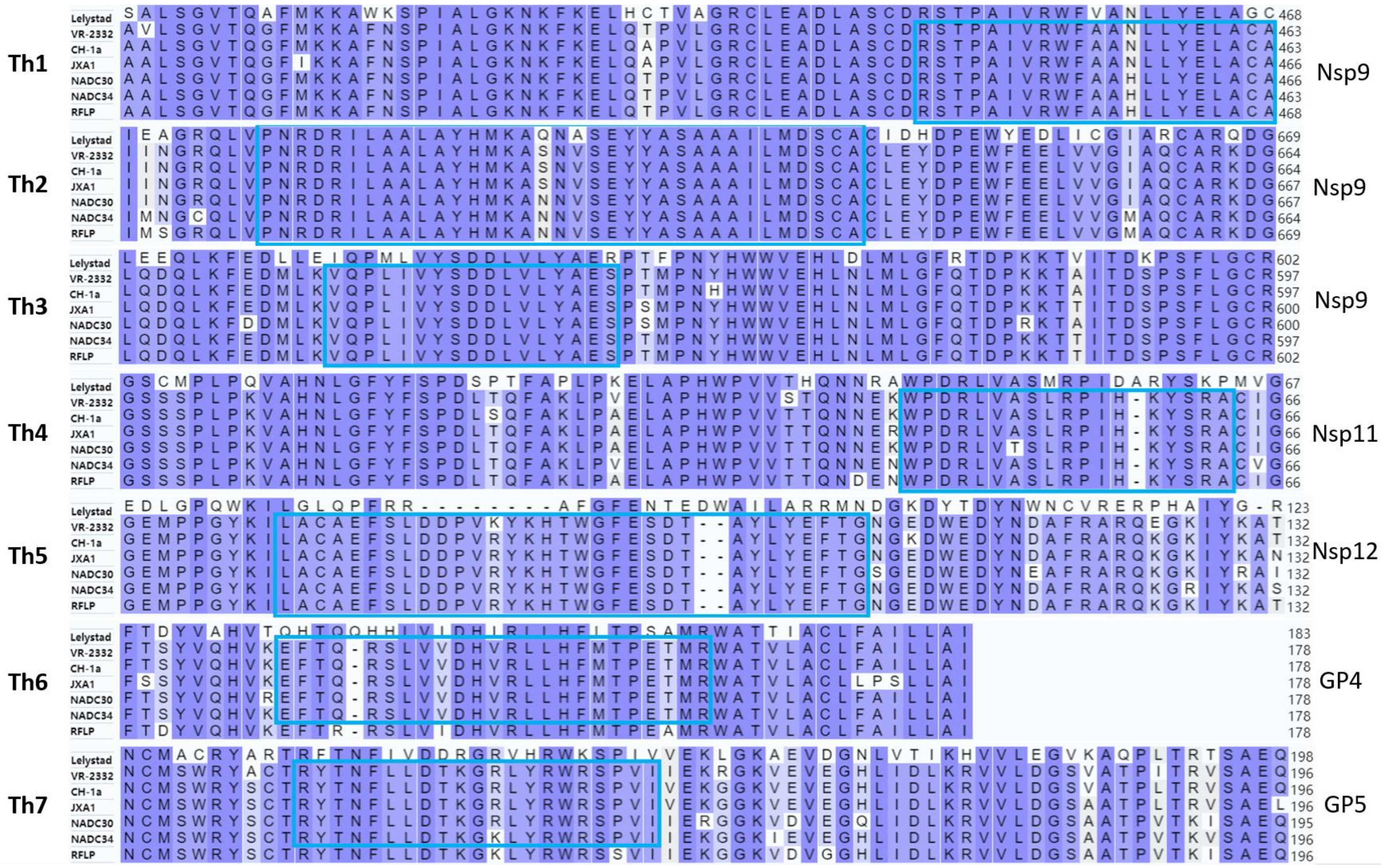

Figure S1 Localization of Th epitopes within PRRSV-encoded proteins, along with sequence comparisons of epitopes across seven representative strains.

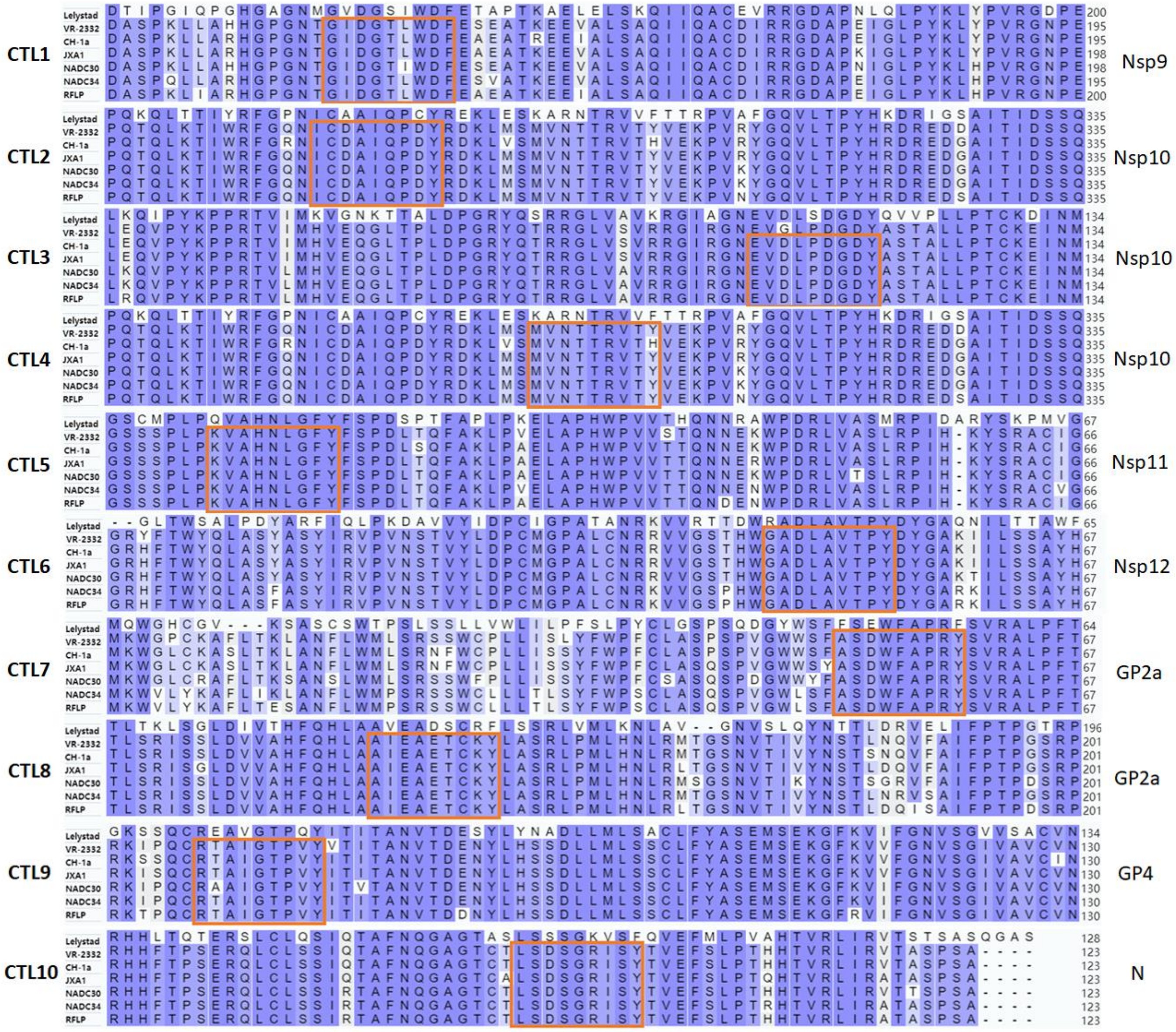

Figure S2 Localization of CTL epitopes within PRRSV-encoded proteins, along with sequence comparisons of epitopes across seven representative strains.

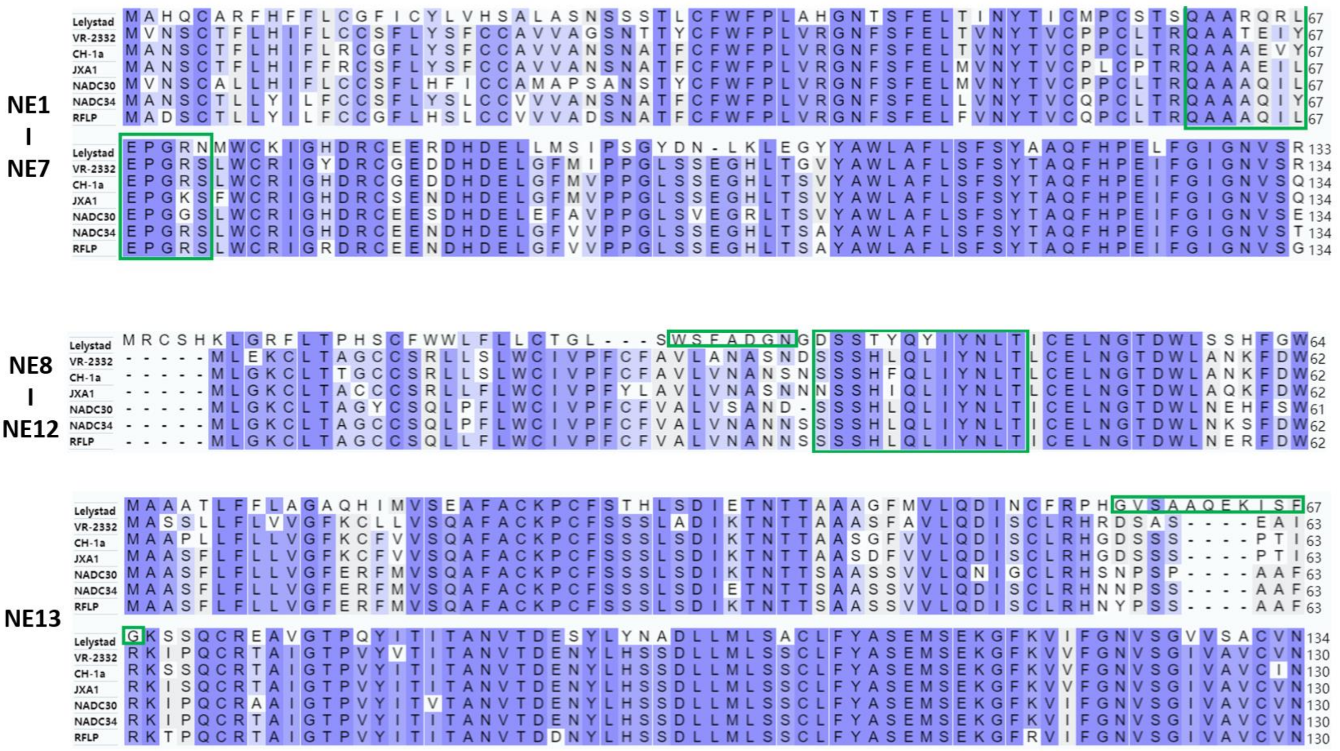

Figure S3 Localization of neutralizing epitopes within PRRSV-encoded proteins, along with sequence comparisons of epitopes across seven representative strains.

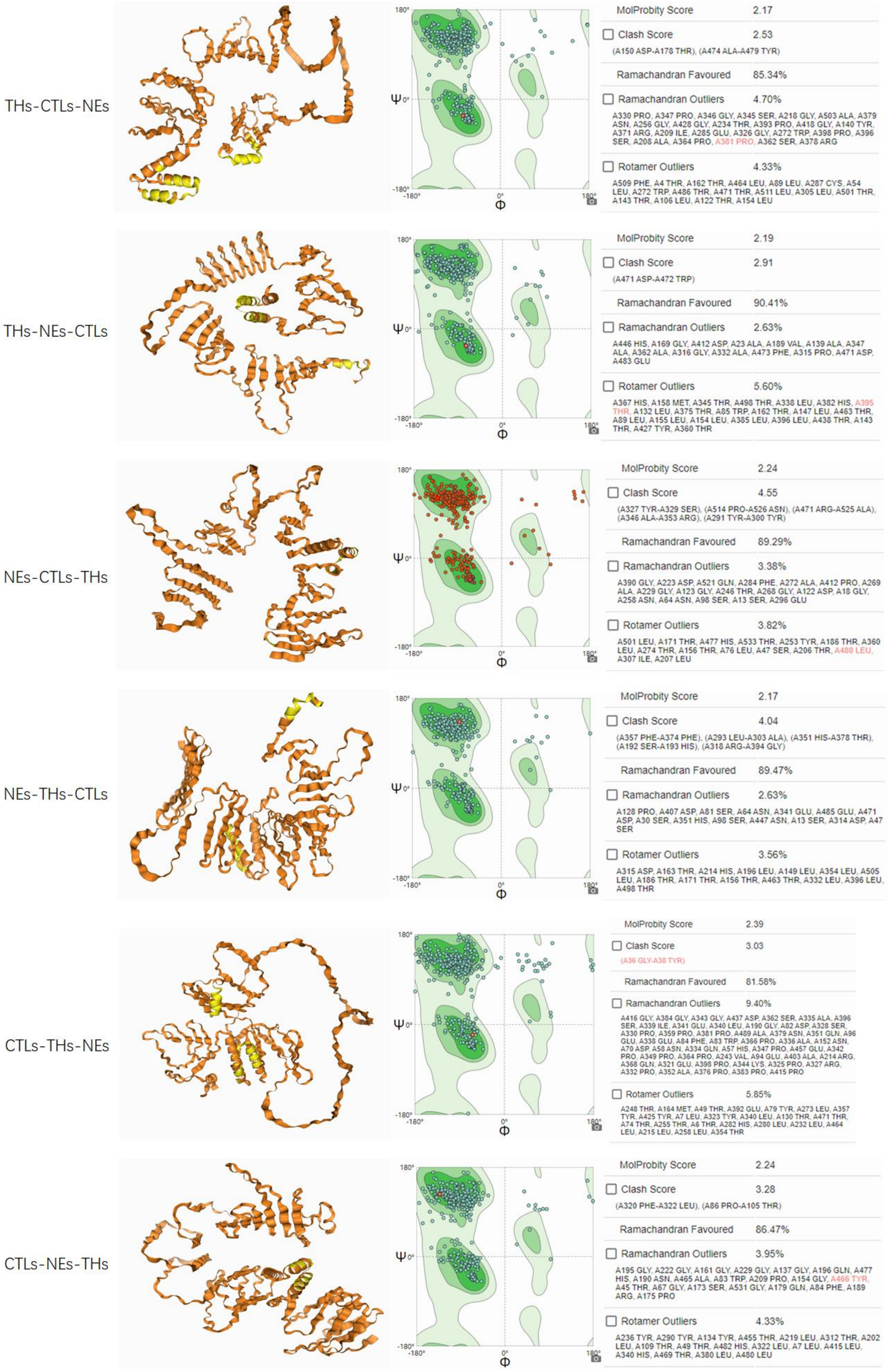

Figure S4 Structural simulation and assessment of six THs, CTLs, and NEs concatenation arrangements.

Table S4. Receptor-ligand interface residue pairs of TLR4 and THs-CTLs-NEs protein complex.

Receptor residue	Ligand residue	Distance (Å)	Receptor residue	Ligand residue	Distance (Å)	Receptor residue	Ligand residue	Distance (Å)
27A	465A	4.591	47A	463A	5.000	103A	277A	2.651
27A	467A	2.154	50A	429A	4.589	103A	293A	2.149
27A	469A	4.657	50A	430A	3.116	105A	236A	2.733
28A	153A	2.643	51A	430A	3.668	105A	271A	3.466
28A	465A	2.923	51A	467A	3.881	106A	236A	1.900
28A	467A	4.292	52A	430A	3.869	127A	271A	4.777
29A	153A	4.851	54A	429A	4.433	127A	276A	2.403
30A	153A	4.540	54A	430A	4.227	129A	236A	3.422
30A	155A	4.913	57A	239A	2.965	129A	271A	4.808
30A	176A	3.164	57A	269A	3.760	129A	272A	2.866
33A	231A	3.718	57A	277A	2.608	130A	161A	4.750
36A	239A	2.062	58A	238A	3.775	130A	235A	4.186
37A	239A	3.989	58A	239A	3.276	130A	236A	3.380
39A	155A	4.236	60A	157A	4.029	134A	164A	3.649
39A	157A	2.571	63A	121A	2.660	153A	272A	0.769
41A	155A	2.393	63A	122A	4.147	156A	164A	4.343
41A	157A	3.678	63A	123A	2.710	158A	164A	2.452
42A	120A	2.230	64A	121A	4.050	159A	164A	3.627
42A	121A	2.192	65A	121A	3.119	178A	272A	4.046
42A	122A	3.105	75A	428A	3.429	181A	164A	3.466
42A	123A	3.139	76A	428A	4.241	183A	164A	3.010
42A	124A	3.018	78A	277A	4.530	184A	164A	4.798
43A	120A	4.012	78A	293A	4.595	234A	164A	4.622
43A	121A	4.199	79A	277A	1.589	234A	165A	4.910
43A	153A	2.969	79A	293A	4.659	234A	166A	3.158
44A	120A	3.039	81A	236A	4.914	263A	164A	3.303
44A	121A	3.533	81A	238A	4.393	263A	166A	4.690
44A	463A	2.515	81A	269A	3.891	264A	97A	2.858
45A	462A	4.931	81A	270A	4.775	264A	99A	3.163
45A	463A	4.235	81A	271A	3.493	264A	192A	2.664
45A	465A	4.636	81A	277A	4.193	265A	97A	3.663
46A	461A	4.518	82A	236A	4.543	291A	166A	4.815
46A	462A	1.026	87A	122A	4.904	292A	166A	4.914
46A	463A	2.910	89A	121A	4.819	316A	166A	4.762
46A	492A	3.806	102A	276A	3.658	317A	166A	4.640
47A	460A	3.656	102A	293A	4.277	339A	192A	3.786
47A	461A	2.974	103A	271A	3.108			
47A	462A	4.561	103A	276A	3.264			

Table S5. Receptor-ligand interface residue pairs of TLR2 and THs-CTLs-NEs protein complex.

Receptor residue	Ligand residue	Distance (Å)	Receptor residue	Ligand residue	Distance (Å)	Receptor residue	Ligand residue	Distance (Å)
20B	239A	4.427	56B	124A	3.399	509B	515A	2.433
20B	241A	4.540	71B	236A	2.709	509B	516A	2.135
20B	265A	3.886	71B	272A	4.200	509B	517A	2.373
21B	239A	3.400	74B	160A	3.496	509B	520A	4.127
21B	267A	3.254	74B	164A	3.966	530B	517A	2.798
21B	279A	4.522	74B	235A	4.944	530B	520A	3.860
21B	289A	4.987	75B	233A	2.886	530B	521A	3.261
22B	239A	4.466	75B	234A	4.679	530B	524A	2.653
23B	239A	3.644	75B	235A	3.116	532B	520A	3.797
23B	269A	3.754	75B	236A	4.857	533B	520A	4.178
24B	277A	4.247	77B	158A	3.106	553B	534A	3.814
24B	289A	3.377	77B	159A	3.841	557B	524A	2.818
24B	291A	3.729	77B	160A	2.449	558B	520A	4.158
25B	276A	3.942	78B	123A	2.751	558B	524A	3.514
25B	277A	3.827	78B	156A	3.755	559B	534A	3.957
27B	429A	4.281	78B	158A	4.583	560B	533A	4.791
28B	429A	4.594	80B	121A	3.323	560B	534A	3.760
33B	153A	2.666	80B	122A	1.838	561B	513A	4.034
34B	120A	4.487	80B	123A	4.281	561B	515A	4.718
34B	121A	3.708	98B	164A	4.485	561B	532A	2.213
34B	153A	4.449	99B	160A	3.761	561B	533A	4.082
36B	121A	4.528	175B	1A	3.505	562B	513A	3.834
41B	429A	4.835	176B	1A	3.375	562B	515A	3.711
42B	429A	3.501	176B	2A	3.233	562B	520A	2.578
46B	276A	3.865	178B	2A	3.192	564B	511A	4.956
52B	157A	3.454	199B	1A	3.804	564B	512A	3.316
53B	123A	3.074	200B	1A	2.585	564B	513A	4.559
53B	156A	4.568	431A	424A	2.287	564B	514A	4.393
53B	157A	3.274	454A	422A	4.767	571B	510A	4.448
53B	158A	3.960	486B	517A	4.149	572B	480A	3.786
54B	123A	3.175	487B	516A	4.771	572B	510A	2.743
54B	155A	4.608	487B	517A	3.851	572B	512A	4.681
54B	157A	3.059	495A	419A	2.990	573B	510A	2.553
55B	123A	4.585	497A	419A	3.149	573B	511A	4.667
56B	120A	2.741	498A	419A	2.571	573B	512A	2.527
56B	121A	2.654	506B	521A	2.870	574B	478A	4.826
56B	122A	4.712	508B	517A	2.728	574B	508A	3.164
56B	123A	3.328	508B	520A	3.662			
